# Preventing Sudden Cardiac Death in Mitral Valve Prolapse: When Multimodality Imaging Is the Key to Success

**DOI:** 10.3390/jcm11175112

**Published:** 2022-08-30

**Authors:** Anna Giulia Pavon, Luca Bergamaschi, Marco Guglielmo

**Affiliations:** 1 Division of Cardiology, Cardiocentro Ticino Institute, Ente Ospedaliero Cantonale (EOC), Via Tesserete 48, 6900 Lugano, Switzerland; 2 Unit of Cardiology, IRCCS Policlinico St. Orsola-Malpighi, Department of Experimental, Diagnostic and Specialty Medicine-DIMES, University of Bologna, 40138 Bologna, Italy; 3 Department of Cardiology, Division of Heart and Lungs, University Medical Center Utrecht, Utrecht University, 3584 CX Utrecht, The Netherlands

Mitral valve prolapse (MVP) is a common cardiac anomaly that is estimated to affect 1–3% of the general population [1]. Firstly described by Barlow et al. [2] in 1968, this entity has a very heterogeneous clinical spectrum from being completely asymptomatic to complex ventricular arrhythmia (cVA) and sudden cardiac death (SCD) [3]. Overall, the estimated risk of malignant arrhythmic events in subjects with MVP has been reported from 0.2% to 0.4% (between 16 and 41 per 10,000 per year) [3]. Despite the incidence appearing to be relatively low, it is 3-fold higher than the risk of SCD in the general population (0.1% per year) [3,4]. In a landmark paper, Basso et al. highlighted how in subjects <35 years, MVP was the third cardiological cause associated with SCD, with an incidence of 12% [5]. Therefore, since MVP can affect a larger percentage of population, it appears of detrimental importance to stratify the arrhythmic risk and to identify patients that may benefit from deeper investigation or specific treatment. In this context cardiac imaging plays a pivotal role in identifying features associated with higher risk of cVA and SCD. 

Transthoracic echocardiography (TTE) is the first-line imaging tool to evaluate MVP, which is classically defined as a superior displacement of the mitral leaflet(s) of >2 mm during systole and myxomatous degeneration of the mitral leaflets, resulting in a maximum leaflet thickness of at least 5 mm during diastasis [6]. When performing TTE in the presence of MVP, attention must be paid to such cases of anatomical features as bi-leaflet MVP [4] and mitro-annular disjunction (MAD) [5]; the latter is defined as the systolic separation of the mitral leaflet insertion from the LV myocardium [5]. Additionally, the unusual systolic motion of the posterior mitral ring on the adjacent myocardium (the so-called “Curling effect”) [7] and the evidence of a spiked lateral systolic velocity ≥16 cm/s (“Pickelhaube’s sign”) in Tissue Doppler are associated with cVA and SCD and should be included in the routine echocardiographic evaluation of MVP patients [8]. 

Recent studies have highlighted how the evaluation of myocardial composition with Cardiovascular Magnetic Resonance (CMR) is of detrimental importance in the arrhythmic risk stratification of patients with MVP [9]. Surely the identification of MAD can be easier in CMR compared to TTE, as documented by its high specificity (96%) [10].

Notably, the concept of MAD was first introduced by Bharati et al. in 1981 [11], and the largest cohort study published so far considering the prognosis of MAD has shown that its presence is independently associated with long-term incidence of clinical arrhythmic events, but not with an increased risk of mortality [5]. It must be pointed out that to date, a specific pathomorphological definition of MAD is still lacking, and anatomical findings of Angelini et al. [12] and Toh et al. [13] show how MAD appears to be a common feature of normal adult heart. Based on these findings, the hypothesis of two patterns of MAD has recently been created [14]. A “pseudo-MAD”, in which the MAD is only detected in systole with the juxtaposition of the belly of the billowing posterior leaflet on the adjacent left atrial wall, giving the illusion that a disjunction is present, but a normal attachment of the leaflet can be observed in the diastolic phase, and a “true-MAD”, when the disjunction can be seen in both systole and diastole and it is linked to an abnormal attachment of the leaflet in the atrial wall. Certainly, further studies to validate this hypothesis and to correlate it with the risk of arrhythmias are needed, but this aspect brings back to the fore the need for deeper knowledge on myocardial composition in patients with MVP. 

Actually, the presence of macroscopic myocardial fibrosis in Late Gadolinium Enhancement (LGE) sequences in Cardiovascular Magnetic Resonance (CMR) has been correlated to a high risk of cVA and SCD in several imaging studies [5,7,15] and in histopathologic analyses of young SCD victims with MVP [7]. Those myocardial alterations are typically located in the inferior, infero-lateral wall and at the level of papillary muscles, probably as a result of the mechanical stretch acting upon the valve and could represent the arrhythmic substrate in patients with MVP [7]. To note, the identification of LGE at the PM level and neighboring LV walls may be challenging even for CMR experts, and additional non-standard views as well as the use of novel imaging technique may be needed to best visualize fibrotic regions in the myocardium [16]. Moreover, the precise detection of fibrosis using LGE may be challenging in areas where the myocardium is not completely replaced by fibrotic tissue [17].

It appears clear that myocardial composition has a fundamental role in arrhythmic risk stratification so that the scientific community is actively working in detecting other possible early risk stratification markers. In this setting, the possible role of interstitial fibrosis in arrhythmogenesis has been evoked. Actually, the presence of elevated native T1 values and higher values of extracellular volume, associated with the presence of interstitial fibrosis, have been reported in several studies, suggesting that the fibrotic alterations in patients with MVP may go far beyond the presence of macroscopic fibrosis detected by LGE [1,18,19,20]. However, to date, only retrospective and relatively small studies have been published so far, and all of them failed to demonstrate a direct role of interstitial fibrosis in arrhythmogenesis, which needs further evaluation in larger populations.

Other than T1 mapping, advanced functional imaging parameters could also be useful for the detection of subclinical myocardial structural changes in patients with MVP. As is widely known, myocardial strain assessed by either speckle-tracking echocardiography or by feature-tracking CMR evaluation (CMR-FT) is a sensitive marker of cardiac dysfunction, with proven long-term prognostic value in many cardiac conditions [21,22]. In particular, CMR-FT is able to identify abnormalities in left ventricular deformation even in the presence of normal parameters of systolic function [23]. In the early stages of the disease, the mechanical stretch induced by the systolic tugging (‘curling’) present in the MVP determines higher strain values in the papillary muscles but also in the prolapsed scallops of the mitral valve, as evaluated in a brilliant 3D-echocardiography study using valve strain quantification in MVP patients [24]. Subsequently, the constant damaging effect of MVP stretching and the resulting incoming interstitial fibrosis led to reduced strain values in these regions. In fact, a recent study showed that MVP patients with moderate to severe mitral regurgitation exhibit lower CMR-FT and combined higher T1 mapping values, especially in the basal and mid-LV inferolateral walls, compared to normal controls [24]. However, even though this is promising, the role of deformation parameters in arrhythmic risk stratification needs further larger studies.

In conclusion, it must be noted that arrhythmogenesis in patients with mitral valve prolapse is still not completely understood. To date, no specific guidelines are available to evaluate the risk stratification, treatment, and follow-up in patients with arrhythmic MVP; however, it is clear how a comprehensive evaluation based on multimodality imaging is of detrimental importance to identify the minority of patients with high-risk features for whom a more aggressive management strategy might be considered [25], including pharmacological treatment, catheter ablation procedures [26], implantable cardioverter defibrillator implantation, or mitral valve surgery [27].
